# A case of SARS-CoV-2 Omicron reinfection resulting in a significant immunity boost in a paediatric patient affected by B-cell acute lymphoblastic leukemia

**DOI:** 10.1186/s12879-023-08111-4

**Published:** 2023-03-07

**Authors:** Rossana Scutari, Valeria Fox, Maria Antonietta De Ioris, Vanessa Fini, Annarita Granaglia, Valentino Costabile, Luna Colagrossi, Cristina Russo, Angela Mastronuzzi, Franco Locatelli, Carlo Federico Perno, Claudia Alteri

**Affiliations:** 1 Department of Oncology and Hemato-Oncology, University of Milan, Milan, Italy; 2 Multimodal Research Area, Unit of Microbiology and Diagnostics in Immunology, Bambino Gesù Children’s Hospital, IRCCS, Rome, Italy; 3 Department of Pediatric Hematology/Oncology and Cellular and Gene Therapy, Bambino Gesù Children’s Hospital IRCCS, Rome, Italy

**Keywords:** Omicron reinfection case report, BA.1, BA.2, SARS-CoV-2, Immunocompromised paediatric patient

## Abstract

**Background:**

Since its emergence in November 2021, SARS-CoV-2 Omicron clade has quickly become dominant, due to its increased transmissibility and immune evasion. Different sublineages are currently circulating, which differ in mutations and deletions in regions of the SARS-CoV-2 genome implicated in the immune response. In May 2022, BA.1 and BA.2 were the most prevalent sublineages in Europe, both characterized by ability of evading natural acquired and vaccine-induced immunity and of escaping monoclonal antibodies neutralization.

**Case presentation:**

A 5-years old male affected by B-cell acute lymphoblastic leukemia in reinduction was tested positive for SARS-CoV-2 by RT-PCR at the Bambino Gesù Children Hospital in Rome in December 2021. He experienced a mild COVID-19 manifestation, and a peak of nasopharyngeal viral load corresponding to 15.5 Ct. Whole genome sequencing identified the clade 21 K (Omicron), sublineage BA.1.1. The patient was monitored over time and tested negative for SARS-CoV-2 after 30 days. Anti-S antibodies were detected positive with modest titre (3.86 BAU/mL), while anti-N antibodies were negative. 74 days after the onset of the first infection and 23 days after the last negative test, the patient was readmitted to hospital with fever, and tested positive for SARS-CoV-2 by RT-PCR (peak of viral load corresponding to 23.3 Ct). Again, he experienced a mild COVID-19. Whole genome sequencing revealed an infection with the Omicron lineage BA.2 (21L clade). Sotrovimab administration was started at the fifth day of positivity, and RT-PCR negativity occurred 10 days later. Surveillance SARS-CoV-2 RT-PCR were persistently negative, and in May 2022, anti-N antibodies were found positive and anti-S antibodies reached titres > 5000 BAU/mL.

**Conclusions:**

By this clinical case, we showed that SARS-CoV-2 reinfection within the Omicron clade can occur and can be correlated to inadequate immune responses to primary infection. We also showed that the infection’s length was shorter in the second respect to first episode, suggesting that pre-existing T cell-mediated immunity, though not preventing re-infection, might have limited the SARS-CoV-2 replication capacity. Lastly, Sotrovimab treatment retained activity against BA.2, probably accelerating the viral clearance in the second infectious episode, after which seroconversion and increase of anti-S antibodies titres were observed.

**Supplementary Information:**

The online version contains supplementary material available at 10.1186/s12879-023-08111-4.

## Background

Risk of SARS-CoV-2 reinfection has been widely described since the first phases of the pandemic, regardless of the community infection rates, and mainly in immunocompromised individuals. This fragile population is indeed particularly susceptible to flare and lack of response to vaccination due to impaired humoral immunity and ability to produce neutralizing antibodies [1–4].

While no change in the SARS-CoV-2 reinfection risk was observed in the general as well as in paediatric population throughout all the first epidemic waves [5], the frequency of SARS-CoV-2 reinfection cases has started to significantly increase concomitantly with the emergence of the Omicron variant [6].

SARS-CoV-2 Omicron lineage was first reported in November 2021 in South Africa and quickly spread, immediately becoming the dominant lineage worldwide [7, 8]. This lineage is characterised by several mutations in key regions of the SARS-CoV-2 genome, as well as RDB domain in the spike protein, that confer to Omicron increased transmissibility and escape against naturally acquired, vaccine-induced immunity, and mAbs treatment [9–11]. BA.1 and BA.2 were the most prevalent sublineages characterizing Omicron clade in Europe since May 2022 [12]. These 2 lineages do not differ for infectivity and neutralization sensitivity to omicron-infected and vaccinated patients’ sera, [13, 14] notwithstanding some amino acid mutations and deletions occurring in SARS-CoV-2 spike protein differentiated them [15]. Most of mAbs lost their efficacy against all Omicron lineages, and initial in vitro data reports a 27-fold decrease in the neutralizing activity of Sotrovimab against the BA.2 respect to BA.1 [16], suggesting a potentially reduced efficacy of Sotrovimab against BA.2 and forthcoming Omicron lineages.

Here we describe a case of Omicron intra-clade reinfection efficiently resolved after Sotrovimab administration in a patient affected by a B-cell acute lymphoblastic leukaemia. Whole genome sequencing of samples from the two distinct infection episodes revealed the presence of BA.1 and BA.2 sublineages of the Omicron clade.

## Case presentation

**Fig. 1:**
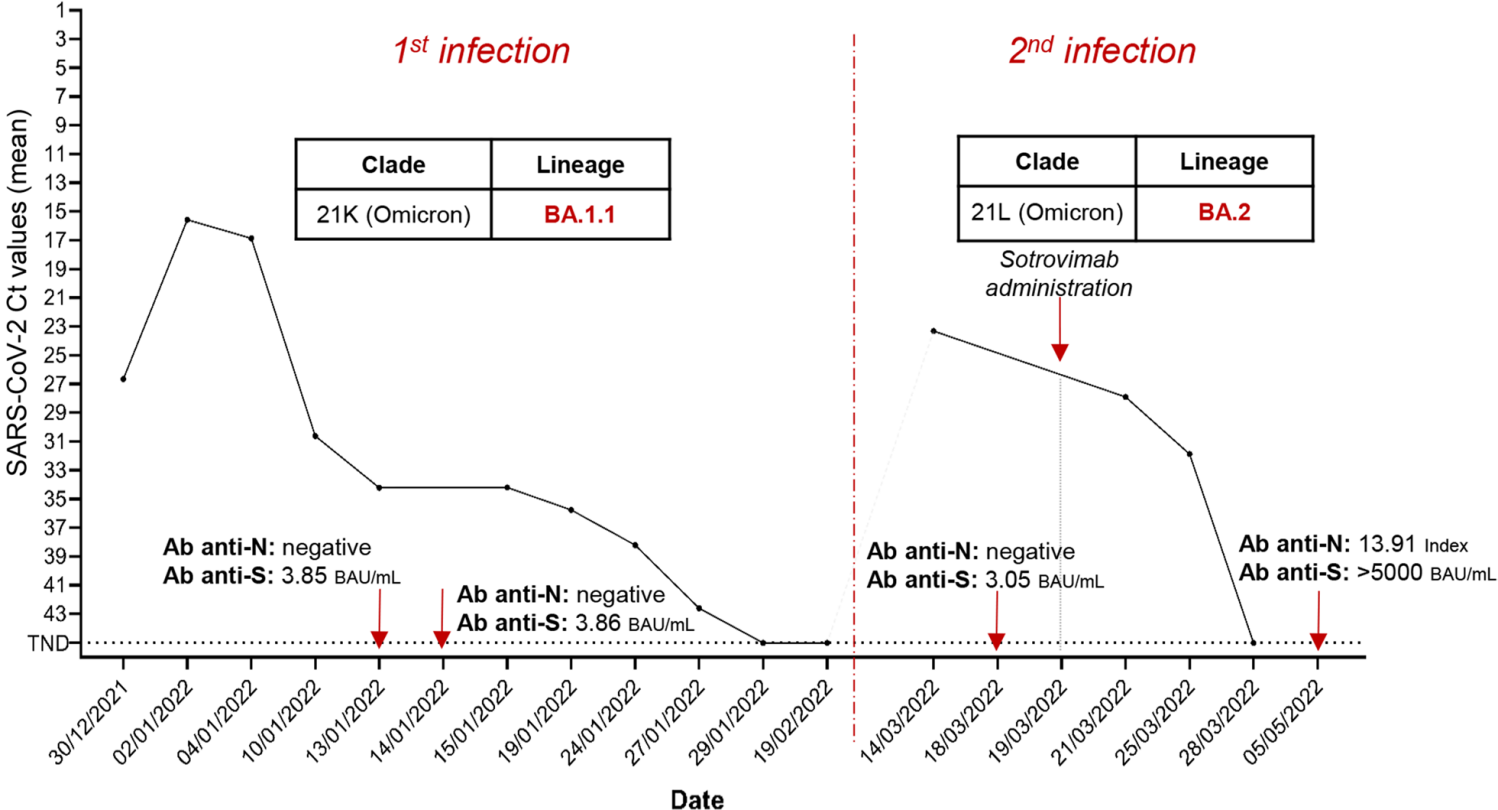
SARS-CoV-2 viral load expressed as mean Ct values obtained by real time PCR over time. The dotted line indicates the SARS-CoV-2 undetectability. *CT* cycle threshold values, *RT-PCR* real-time polymerase chain reaction, *TND* target not detected, *Ab* antibodies

**Fig. 2:**
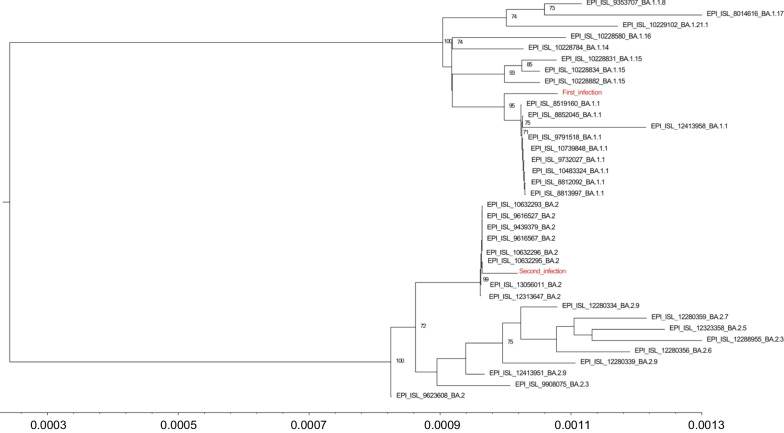
Phylogenetic tree. Estimated maximum likelihood (ML) phylogeny of SARS-CoV-2 genomes from two samples of the same patient (red). Representative Omicron BA.1 and BA.2 genomes retrieved by GISAID (n = 34) (black) were also included. The ML phylogeny was estimated with IqTree using the best-fit model of nucleotide substitution TrN + I + G4 with 1000 replicates fast bootstrapping

A 5-years old Caucasian male patient affected by B-cell acute lymphoblastic leukaemia (Table 1) treated according to the AIEOP BFM Protocol ALL 2017 (Protocol Identifier: NCT03643276) [17] was admitted at the Bambino Gesù Children Hospital IRCCS in Rome in December 2021 and was tested negative to SARS-CoV-2 by RT-PCR in the context of hospital screening (Additional file 1: Figure S1). The patient was not vaccinated against SARS-CoV-2. After 4 days from admission, he started to manifest fever and was subjected to a nasopharyngeal swab, which resulted positive to SARS-CoV-2 with a mean Ct value of 26.65 by RT-PCR (Fig. 1 and Additional file 2: Table S1) (day 1 of SARS-CoV-2 infection). The patient experienced a mild COVID-19 with fever and without symptoms at lower respiratory airways, except for a peribronchial thickening observed at the chest X-rays. The viral load peak was observed at day 4, when the mean Ct value was 15.55 (Fig. 1 and Additional file 2: Table S1). SARS-CoV-2 whole genome sequencing revealed the presence of clade 21 K (Omicron), sublineage BA.1.1 (Figs. 1 and 2). The patient continued to test positive at the nasopharyngeal swab by RT-PCR until day 31, when the RT-PCR resulted negative (Fig. 1 and Additional file 2: Table S1). At days 15 and 16, the antibodies against SARS-CoV-2 spike (anti-S) and nucleocapsid (anti-N) were monitored, revealing low levels of anti-S (3.85 and 3.86 BAU/mL, respectively; cut-off < 0.8) and absence of anti-N (Fig. 1). At day 52, 3 weeks after the RT-PCR negative result, a nasopharyngeal swab antigenic test was performed, confirming again the negativity to SARS-CoV-2.

**Table 1:** Demographic and clinical characteristics

Demographic characteristics				
Age	Gender	Origin	Comorbidities	SARS-CoV-2 Vaccination
5	Male	Caucasian	B-cell acute lymphoblastic leukemia in reinduction	No

Clinical characteristics		
	First infection	Second infection
First positivity	30/12/2021	14/03/2022
Nasopharyngeal swab sample date	04/01/2022	14/03/2022
Length of SARS-CoV-2 positivity	30 days	15 days
Fever	+	+
COVID-19 definition		
Asymptomatic	−	−
Mild^a^	+	+
Moderate/severe^b^	−	−

^a^Including: symptoms of upper respiratory airways (cough, sore throat, runny nose, sneezing, rhinitis, pharyngo-adenitis, laryngitis)

^b^Including: symptoms of lower respiratory airways (pneumonia, bronchitis and bronchiolitis)

After 74 days since the onset of the first infection and 23 days after the last negativity, the patient was re-admitted at the hospital with fever. At day 1, a nasopharyngeal swab resulted positive for SARS-CoV-2 by RT-PCR, with a mean Ct value of 23.3 (Fig. 1 and Additional file 2: Table S1). The SARS-CoV-2 whole genome sequencing revealed an infection with the Omicron BA.2 sublineage, clade 21L (Figs. 1 and 2). At day 5, antibody testing revealed low levels of anti-S (3.05 BAU/mL; cut-off < 0.8), whereas no anti-N antibodies were detected (Fig. 2). As in the first episode, the patient experienced a mild infection, and exclusively reported fever and upper respiratory airways symptoms. At day 1, he presented low neutrophils counts and G-CSF was administered since day 4. At day 6, Sotrovimab was administered according to hospital’s policy [18] and its higher reported efficacy against Omicron BA.2 lineage compared to the combinations bamlanivimab plus etesevimab and casirivimab plus imdevimab [19]. Nasopharyngeal swabs were repeated at days 8 and 10, resulting in a progressive drop of viral load (mean Ct: 27.88 and 31.85, Fig. 1 and Additional file 2: Table S1).

After 15 days since the onset of symptoms, the patient resulted negative to a nasopharyngeal antigenic test. Thirty-eight days after negative antigenic test, patient was subjected to serological tests that revealed high levels of anti-S (> 5000 BAU/mL; cut-off < 0.8) and anti-N antibodies (13.91 Index).

For details regarding material and methods refer to Additional file 3.

## Discussion and conclusions

The case here described was categorized as a reinfection according to the US Center for Disease Control and Prevention (CDC) criteria for reinfection [20] and to the European Center for Disease Prevention and Control (ECDC) criteria [21]. In particular, both infections were mild symptomatic, and the second infection occurred 74 days after the onset of the first infection. Respiratory specimens were available for both episodes at different time points and every episode had at least one associated positive RT-PCR test.

The first infection, due to the BA.1.1 sublineage, was characterised by high viral load, and a rather long positivity (30 days). Final negativity was confirmed by two different tests at different time-points. Serological data revealed that anti-N antibodies were absent during and after the first infection, while the level of anti-S antibodies remained low. Low levels of anti-S and no anti-N antibodies were indeed evident at the day 5 of the second infection. These data confirm that the patient developed little or no detectable antibodies after the first infection, consistent with a failure in mounting an effective protective immunity, and thus associated to increased risk of reinfection [1–4]. These findings underlie the importance of vaccination in immunocompromised patients, to both elicit an immune response in those who responded to the primary infection, and to induce a de novo response in those who did not mount a detectable antibody response during primary infections [22].

Beyond the immune status, other factors may influence the risk of re-infection, like the spread of genetically distinct lineage of SARS-CoV-2, characterized by several non-synonymous mutations in the S region, that could lead to immune evasion [9–12]. At this regard, even if the sublineage BA.2 is not associated with an increased transmissibility and immune evasion than the lineage BA.1 [13, 14]*,* reinfections by the BA.2 shortly after a previous BA.1 infection have been recently described, mostly in not vaccinated, young individuals [23]. Consistent with these findings, our clinical case was a SARS-CoV-2 unvaccinated immunocompromised child, experiencing a BA.2 reinfection after a previous BA.1.1 infection. Second infection was characterized by a shorter period of positivity (15 days) compared to the first infection. In absence of a few viral load quantification tests in the days of second infection and before Sotrovimab administration, we cannot speculate about a lower peak of viral load in the second infection respect to the first infection, but the shorter duration (15 days) of the second infection compared to the first (30 days) might indicate a more superficial and transient secondary infection. Of note, the rapid viral load drop observed in the second infection could be driven by the T cell-mediated immunity obtained during the first infection [24] supporting the hypothesis that the first infection might have acted by ‘priming’ the immune system. This drop could also have been accelerated by Sotrovimab administration at the beginning of the second episode. Although a decrease in the neutralizing activity of Sotrovimab against the BA.2 sublineage is suggested [16], in this clinical case Sotrovimab treatment retains activity. After the second infection, anti-N seroconversion and increase of anti-S titres were also observed. Interestingly, Sotrovimab administration has been recently suggested to enhance serum SARS-CoV-2 S antibody levels in patients infected with the SARS-CoV-2 Omicron clade [25]. Thus, T cell-mediated immunity and Sotrovimab administration might result in additive or synergistic effect able to finally promote the resolution of the second infection.

In conclusion, by this clinical case we showed that SARS-CoV-2 reinfection within the Omicron clade can occur and can be correlated to inadequate immune responses to primary infection. We also showed that nasopharyngeal viral load was lower in the second respect to first episode, suggesting that pre-existing T cell-mediated immunity, though not preventing re-infection, might have limited the nasopharyngeal viral load. Sotrovimab treatment also retained activity against BA.2, probably accelerating the viral clearance in the second infectious episode, after which seroconversion and increase of anti-S antibodies titres were observed. Lastly, our study highlights the importance of a full vaccination strategy in immunosuppressed individuals for the prevention of SARS-CoV-2 infection and for the mounting of an adequate immune response against viral replication.

## Supplementary Information

**Additional file 1: Figure S1. **A timeline of the case. Hospitalization, symptoms, diagnosis test (Antigenic test and RT-PCR); serology, administered treatments, and date of sequencing were reported. ^a^Mild: including symptoms of upper respiratory airways (cough, sore throat, runny nose, sneezing, rhinitis, pharyngo-adenitis, laryngitis). Ab: antibody; CT: Cycle threshold values; RT-PCR: real-time polymerase chain reaction.

**Additional file 2.**

**Additional file 3.**

## Data Availability

The two SARS-CoV-2 sequences characterizing the first and the second infection episode are openly available on GISAID portal under the accession numbers EPI_ISL_13810381 and EPI_ISL_13810380, respectively. All the other data generated or analysed during this study are included in this published article [and its additional information files].

## Abbreviations

BAU: Binding arbitrary unit
CDC: US Center for Disease Control and Prevention
COI: Cut off index
COVID-19: Coronavirus disease 2019
Ct: Cycle threshold
ECDC: European Center for Disease Prevention and Control
ECLIA: Electro-chemiluminescence sandwich immunoassay
G-CSF: Granulocyte colony-stimulating factor
mAbs: Monoclonal antibodies
N: Nucleocapsid
RT-PCR: Real-time reverse transcription polymerase chain reaction
S: Spike
SARS-CoV-2: Severe Acute Respiratory Syndrome Coronavirus 2
