# Supplementary material for: A case of SARS-CoV-2 Omicron reinfection resulting in a significant immunity boost in a paediatric patient affected by B-cell acute lymphoblastic leukemia
Source: BMC Infect Dis. 2023 Mar 7;23:133. doi: 10.1186/s12879-023-08111-4 (PMC9990052; doi:10.1186/s12879-023-08111-4)
Supplement: Supplementary file 1 — Additional file 1: Figure S1. A timeline of the case. Hospitalization, symptoms, diagnosis test (Antigenic test and RT-PCR); serology, administered treatments, and date of sequencing were reported. aMild: including symptoms of upper respiratory airways (cough, sore throat, runny nose, sneezing, rhinitis, pharyngo-adenitis, laryngitis). Ab: antibody; CT: Cycle threshold values; RT-PCR: real-time polymerase chain reaction. [file 12879_2023_8111_MOESM1_ESM.pptx]

## Slide 1
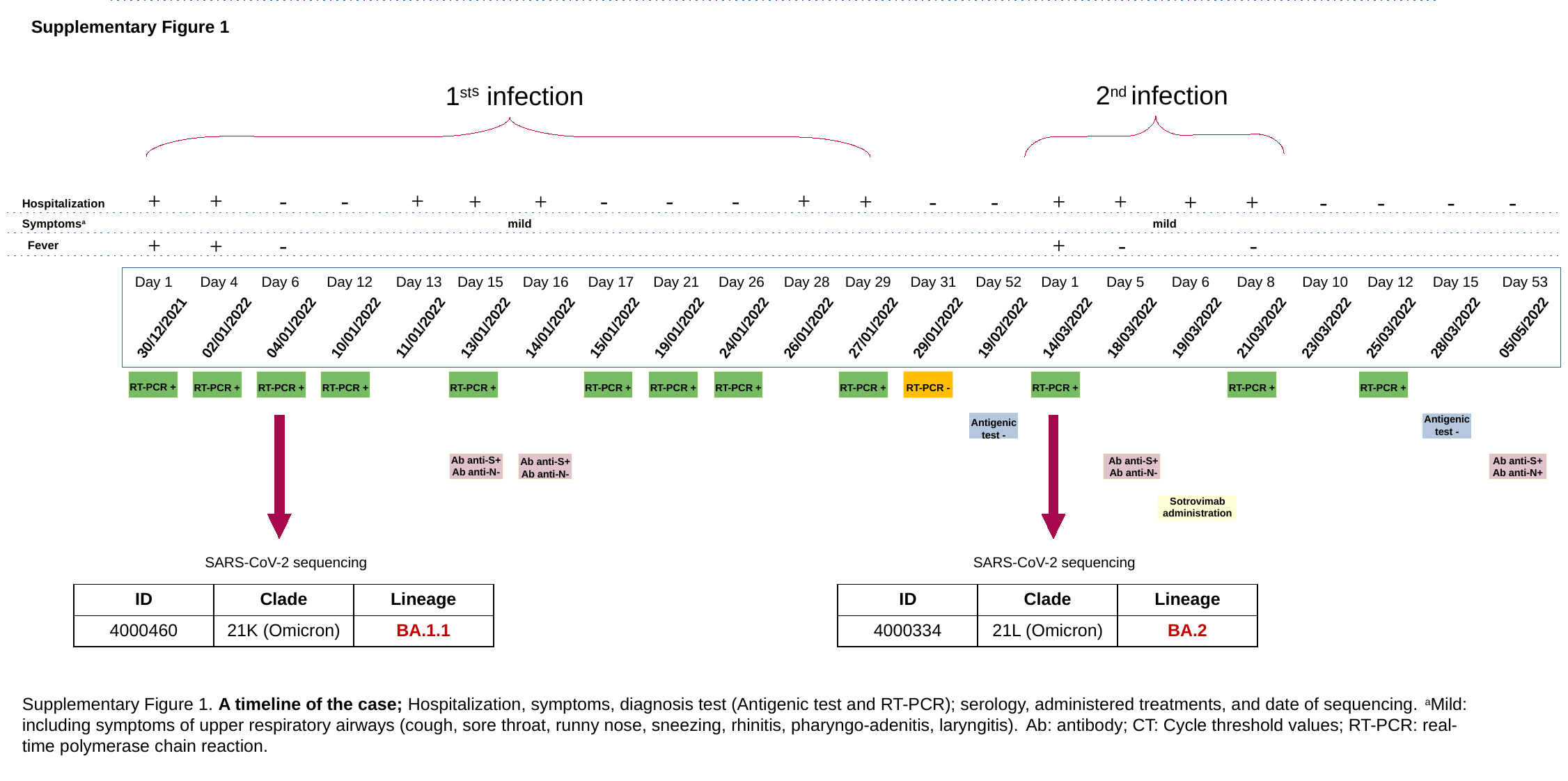

Supplementary Figure 1
2nd infection
1sts infection
+
+
-
-
+
-
-
-
+
+
+
-
-
+
+
+
+
+
-
-
-
-
Hospitalization
Symptomsa
mild
mild
-
+
-
-
+
+
Fever
Day 1
Day 4
Day 6
Day 12
Day 13
Day 15
Day 16
Day 17
Day 21
Day 26
Day 28
Day 29
Day 31
Day 52
Day 1
Day 5
Day 6
Day 8
Day 10
Day 12
Day 15
Day 53
02/01/2022
19/02/2022
23/03/2022
25/03/2022
04/01/2022
13/01/2022
14/01/2022
15/01/2022
24/01/2022
26/01/2022
27/01/2022
19/03/2022
21/03/2022
28/03/2022
05/05/2022
10/01/2022
11/01/2022
19/01/2022
29/01/2022
18/03/2022
30/12/2021
14/03/2022
RT-PCR +
RT-PCR +
RT-PCR +
RT-PCR +
RT-PCR +
RT-PCR +
RT-PCR +
RT-PCR +
RT-PCR +
RT-PCR -
RT-PCR +
RT-PCR +
RT-PCR +
Antigenic test -
Antigenic test -
Ab anti-S+
Ab anti-N-
Ab anti-S+
Ab anti-N-
Ab anti-S+
Ab anti-N+
Ab anti-S+
Ab anti-N-
Sotrovimab
administration
SARS-CoV-2 sequencing
SARS-CoV-2 sequencing
| ID | Clade | Lineage |
| --- | --- | --- |
| 4000460 | 21K (Omicron) | BA.1.1 |
| ID | Clade | Lineage |
| --- | --- | --- |
| 4000334 | 21L (Omicron) | BA.2 |
Supplementary Figure 1. A timeline of the case; Hospitalization, symptoms, diagnosis test (Antigenic test and RT-PCR); serology, administered treatments, and date of sequencing. aMild: including symptoms of upper respiratory airways (cough, sore throat, runny nose, sneezing, rhinitis, pharyngo-adenitis, laryngitis). Ab: antibody; CT: Cycle threshold values; RT-PCR: real-time polymerase chain reaction.
