# Supplementary material for: A case of SARS-CoV-2 Omicron reinfection resulting in a significant immunity boost in a paediatric patient affected by B-cell acute lymphoblastic leukemia
Source: BMC Infect Dis. 2023 Mar 7;23:133. doi: 10.1186/s12879-023-08111-4 (PMC9990052; doi:10.1186/s12879-023-08111-4)
Supplement: Supplementary file 2 — Additional file 2. [file 12879_2023_8111_MOESM2_ESM.docx]

Supplementary Table 1. **Timeline of SARS-CoV-2 RT-PCR and antigenic test results**

|  | | **30/12/2021** | **02/01/2022** | **04/01/2022** | **10/01/2022** | **13/01/2022** | **15/01/2022** | **19/01/2022** | **24/01/2022** | **27/01/2022** | **29/01/2022** | **19/02/2022** | **14/03/2022** | **21/03/2022** | **25/03/2022** | **28/03/2022** |
| --- | --- | --- | --- | --- | --- | --- | --- | --- | --- | --- | --- | --- | --- | --- | --- | --- |
| **Antigenic test (Lumipulse® G SARS-CoV-2 Ag, Fujirebio)** |  |  |  |  |  |  |  |  |  |  |  | Negative |  |  |  | Negative |
| **RT-PCR (Allplex™ SARS-CoV-2 Assay, Seegene)** | **Gene E** |  | 15.18 |  |  |  | 34.55 |  | 38.44 |  |  |  |  | 27.38 | 31.72 |  |
|  | **Gene RdRp/S** |  | 16.50 |  |  |  | 35.13 |  | TND |  |  |  |  | 28.92 | 32.80 |  |
|  | **Gene N** |  | 14.98 |  |  |  | 32.89 |  | 37.94 |  |  |  |  | 27.35 | 31.05 |  |
| **RT-PCR (Xpert® Xpress**  **SARS-CoV-2, Cepheid)** | **Gene N2** |  |  |  |  |  |  |  |  | 42.60 |  |  | 24.20 |  |  |  |
|  | **Gene E** |  |  |  |  |  |  |  |  | TND |  |  | 22.40 |  |  |  |
| **RT-PCR (Simplexa® COVID-19 Direct Kit, DiaSorin Molecular)** | **Gene S** | 26.30 |  | 16.60 | 30.10 | 34.20 |  | 34.70 |  |  | TND |  |  |  |  |  |
|  | **ORF1ab** | 27.00 |  | 17.10 | 31.10 | 34.20 |  | 36.80 |  |  | TND |  |  |  |  |  |
| **RT-PCR** | **Mean Ct** | 26.65 | 15.55 | 16.85 | 30.60 | 34.20 | 34.19 | 35.75 | 38.19 | 42.60 | TND | # | 23.30 | 27.88 | 31.85 | # |

TND: Target Not Detected. RT-PCR: Real-Time reverse transcription polymerase chain reaction.
