# Supplementary material for: A case of SARS-CoV-2 Omicron reinfection resulting in a significant immunity boost in a paediatric patient affected by B-cell acute lymphoblastic leukemia
Source: BMC Infect Dis. 2023 Mar 7;23:133. doi: 10.1186/s12879-023-08111-4 (PMC9990052; doi:10.1186/s12879-023-08111-4)
Supplement: Supplementary file 3 — Additional file 3. [file 12879_2023_8111_MOESM3_ESM.docx]

**Supplementary material**

**Material and Methods**

***Sample collection, and epidemiological data***

This clinical case included all SARS-CoV-2 nasopharyngeal-swabs obtained from December 2021 to May 2022 from a patient aged 5 years affected by B-cell acute lymphoblastic leukemia in reinduction and referred for the diagnosis at Bambino Gesù Children Hospital IRCCS (OPBG) in December 2021. Demographics, epidemiological and clinical data were obtained retrospectively by pseudonymized electronic medical records.

Severity of SARS-CoV-2 infection was defined according to [1], and based on the clinical features, laboratory testing, and chest radiography imaging available for the patient. The following definitions were used: i) asymptomatic infection, defined as children tested SARS-CoV-2 positive after a contact tracing but not developing any clinical symptoms; ii) mild infection, defined as symptoms of upper respiratory tract infection, such as cough, sore throat, runny nose, and sneezing, that may include also fever, fatigue, myalgia, and/or symptoms of gastrointestinal tract infection, defined as vomiting, abdominal pain, nausea or diarrhoea; ii) moderate/severe infection, defined as symptoms of lower respiratory tract infection including clinical signs of bronchitis or pneumonia (fever, cough, dyspnoea, fast breathing) with or without signs of gastrointestinal symptoms.

***SARS-CoV-2 direct detection***

SARS-CoV-2 detection was performed by rapid antigenic tests (Lumipulse® G SARS-CoV-2 Ag, Fujirebio) and Real-time reverse transcription PCR quantification methods (AllplexTM 2019-nCoV Assay, Seegene; Xpert Xpress SARS-CoV-2 Assay, Cepheid; Simplexa® COVID-19 Direct Kit, DiaSorin Molecular) (Supplementary Table 1).

***SARS-CoV-2 serology***

Serum samples were tested on the commercially available Elecsys anti-N and Elecsys anti-S assays (Roche Diagnostics). Antibodies anti-N were expressed in Cut off Index (COI; signal sample/cutoff) and considered as negative if the COI was < 1.0, and as positive if COI was > 1.0 [2,3]. Detection and quantification of anti-S antibodies were automatically calculated in U/mL, equivalent to the Binding Arbitrary Unit (BAU)/mL [4].

***Virus amplification and whole genome sequencing***

Total RNAs were extracted from nasopharyngeal swabs by using QIAamp Viral RNA Mini Kit, followed by purification with Agencourt RNAClean XP beads. Both the concentration and the quality of all isolated RNA samples were measured and checked with the Nanodrop. Virus genomes were generated by using a multiplex approach, using QIAseq DIRECT SARS-CoV-2 Kit, [5] according to the manufacturer’s protocol starting with 50 ng total RNA and followed by Illumina sequencing on a MiSeq system.

Consensus sequences were generated using the GitHub freely distributed software vcf_consensus_builder [6]. All SNPs having a minimum supporting read frequency of 40% with a depth ≥10 were retained.

***Phylogenetic analysis***

SARS-CoV-2 lineages of the two SARS-CoV-2 consensus sequences obtained were assigned according to the PANGOLIN application (Pangolin https://pangolin.cog-uk.io/, v4.0.6) [7]. In order to confirm the sublineages by a phylogenetic approach, 17 BA.1 and 17 BA.2 GISAID deposited sequences selected against homology and sampling date (date ranges: 13/12/2021-27/04/2022), were added. Sequences were aligned using MAFFT (v7.475) and manually inspected in Bioedit. The final alignment was composed of 36 sequences with a length of 29,448 nucleotides. A Maximum-likelihood phylogeny was estimated with IqTree [8], by first inferring the right substitution model (TrN+I+G4) by running ModelFinder [9]. Tree topology was assessed with the fast-bootstrapping function with 1000 replicates. The tree obtained was visualized by using FigTree v1.4.4.

**References**

1. Dong Y, Mo X, Hu Y, Qi X, Jiang F, Jiang Z, Tong S. Epidemiology of COVID-19 Among Children in China. Pediatrics. 2020 Jun;145(6):e20200702. doi: 10.1542/peds.2020-0702.
2. Ko JH, Joo EJ, Park SJ, Baek JY, Kim WD, Jee J, Kim CJ, Jeong C, Kim YJ, Shon HJ, Kang ES, Choi YK, Peck KR. Neutralizing Antibody Production in Asymptomatic and Mild COVID-19 Patients, in Comparison with Pneumonic COVID-19 Patients. J Clin Med. 2020 Jul 17;9(7):2268. doi: 10.3390/jcm9072268.
3. Omata M, Hirotsu Y, Sugiura H, Maejima M, Nagakubo Y, Amemiya K, Hayakawa M, Tsutsui T, Kakizaki Y, Mochizuki H, Miyashita Y. The dynamic change of antibody index against Covid-19 is a powerful diagnostic tool for the early phase of the infection and salvage PCR assay errors. J Microbiol Immunol Infect. 2021 Oct;54(5):830-838. doi: 10.1016/j.jmii.2020.12.009.
4. WHO First WHO International Standard for anti-SARS-CoV-2 immunoglobulin. 2021. https://www.who.int/groups/expert-committee-on-biological-standardization
5. Qiagen. https://www.qiagen.com/us/products/next-generation-sequencing/rna-sequencing/qiaseq-direct-sars-cov-2-kits/
6. Github. https://github.com/peterk87/vcf_consensus_builder. Accessed 12 May 2022.
7. Rambaut A, Holmes EC, O'Toole Á, Hill V, McCrone JT, Ruis C, du Plessis L, Pybus OG. A dynamic nomenclature proposal for SARS-CoV-2 lineages to assist genomic epidemiology. Nat Microbiol. 2020 Nov;5(11):1403-1407. doi: 10.1038/s41564-020-0770-5.
8. Nguyen LT, Schmidt HA, von Haeseler A, Minh BQ. IQ-TREE: a fast and effective stochastic algorithm for estimating maximum-likelihood phylogenies. Mol Biol Evol. 2015 Jan;32(1):268-74. doi: 10.1093/molbev/msu300.
9. Kalyaanamoorthy S, Minh BQ, Wong TKF, von Haeseler A, Jermiin LS. ModelFinder: fast model selection for accurate phylogenetic estimates. Nat Methods. 2017 Jun;14(6):587-589. doi: 10.1038/nmeth.4285.
